# Hyperglycemic memory in the rat bladder detrusor is associated with a persistent hypomethylated state

**DOI:** 10.14814/phy2.14614

**Published:** 2020-11-17

**Authors:** Yi Wang, Moses T. Tar, Kelvin P. Davies

**Affiliations:** ^1^ Department of Urology Albert Einstein College of Medicine Bronx NY USA; ^2^ Department of Physiology and Biophysics Albert Einstein College of Medicine Bronx NY USA

**Keywords:** detrusor, diabetic bladder dysfunction, epigenetics, hyperglycemic memory, metabolism, metabolomics, methylation

## Abstract

Hyperglycemic memory is associated with several complications of diabetes. Although there is some physiological evidence that this phenomenon occurs with diabetic bladder dysfunction (DBD), there have been no studies in bladder that provide evidence of hyperglycemic memory at the molecular/biochemical level. In the present studies, we determined the effects of long‐term diabetes on the metabolome of bladder detrusor in a rat model of streptozotocin‐induced type‐1‐diabetes and the ability of insulin treatment to normalize metabolic changes. These studies demonstrated that although insulin reversed a majority of the metabolic changes caused by diabetes, with long‐term diabetes there was a persistent decrease in the methylation index (indicated by a reduced ratio of S‐adenosylmethionine to S‐adenosyl homocysteine) after insulin treatment. We confirmed a “hypomethylated environment” develops in diabetic detrusor by demonstrating an overall reduction in methylated detrusor DNA that is only partially reversed with glycemic control. Furthermore, we confirmed that this hypomethylated environment is associated with epigenetic changes in the detrusor genome, which are again mostly, but not completely, reversed with glycemic control. Overall our studies provide strong molecular evidence for a mechanism by which diabetes alters methylation status and gene expression in the detrusor genome, and that these epigenetic modifications contribute to hyperglycemic memory. Our work suggests novel treatment strategies for diabetic patients who have attained glycemic control but continue to experience DBD. For example, epigenomic data can be used to identify “actionable gene targets” for its treatment and would also support a rationale for approaches that target the hypomethylation index.

## Graphical abstract

Our studies are the first to provide evidence of “hyperglycemic memory” in the bladder detrusor at the molecular/biochemical level. Evidence is provided that suggests a molecular mechanism for hyperglycemic memory in the diabetic detrusor through epigenetic modification of genomic DNA resulting from an environment of hypomethylation, that persists even after normalization of tissue glucose levels.

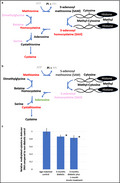

## 1 INTRODUCTION

It is estimated that 34.2 million people suffered from diabetes in the USA in 2018, with 50%–80% of diabetics suffering from lower urinary tract symptoms (LUTS) (Frimodt‐Moller, 1980; Golbidi & Laher, 2010; Lee et al., 2004); LUTS is, therefore, a more common comorbidity than the more widely recognized complications of neuropathy and nephropathy, which affect less than 60% and 50% of diabetics, respectively (Juster‐Switlyk & Smith, 2016; Tuttle et al., 2014). The most common manifestation of LUTS associated with diabetes is diabetic bladder dysfunction (DBD, also known as diabetic cystopathy) (Baneerjee et al., 1985; Kaplan & Blaivas, 1988; Moller, 1976a, 1976b, 1976c; Xiao et al., 2013). Despite advances in techniques for glycemic control, the prevalence rate of DBD in patients receiving treatment remains high, suggesting that DBD is irreversible in some patients (Golbidi & Laher, 2010). In general, studies on patients with type‐1‐diabetes (T1D) suggest that early interventions aimed at glycemic control are more beneficial in treating comorbidities than when applied after prolonged periods of hyperglycemia (Van Den Eeden et al., (2009); Writing Team for the Diabetes Control & Complications Trial/Epidemiology of Diabetes Interventions & Complications Research Group, 2003). This phenomenon has been called hyperglycemic memory and is well‐documented to occur with certain complications of diabetes, such as retinopathy (White et al., 2008), nephropathy (Kowluru et al., 2004), and macrovascular complications (Ceriello, 2012). Epigenetic changes involving the covalent modification of either DNA (by methylation) or histone proteins (by methylation or acetylation) are considered to be major contributing factors to the development of hyperglycemic memory (Al‐Haddad et al., 2016; Villeneuve & Natarajan, 2010).

Previous studies published by our lab provide physiological evidence that hyperglycemic memory occurs in the bladder of diabetic animal models (Melman et al., 2009). These studies report a beneficial effect on bladder pathophysiology in diabetic animal models following insulin treatment, but a return to normal blood glucose levels fails to result in the complete normalization of urodynamic parameters. Although at present there are no published molecular/biochemical peer‐reviewed studies in which hyperglycemic memory has been specifically investigated in the bladder, studies by our lab have identified early‐stage changes in bladder metabolism in a rat model of T1D consistent with those suggested to contribute to mechanisms resulting in hyperglycemic memory in other organs. These included changes in metabolism related to increased oxidative stress and generation of advanced glycation end‐products (Wang et al., 2016). In addition, in the present studies, where we have considered the effects of prolonged hyperglycemia on the metabolome, we observed significant changes in metabolism associated with the methylation of DNA and histones that are involved in epigenetic modification. These involved changes in the levels of S‐adenosylmethionine (SAM) (a methyl‐donor), and s‐adenosyl homocysteine (SAH) and homocysteine (products of the methylation reaction). The ratio of SAM/SAH has been frequently used as an indicator of the cellular methylation index (Stipanuk, 2004) and several studies have associated plasma SAH and homocysteine levels with DNA hypomethylation in other diseases (such as atherosclerosis and vascular disease) (Castro et al., 2003; Esse et al., 2019; Krishna et al., 2010).

In the present studies, we determined the effects of long‐term diabetes on the metabolome of bladder detrusor tissue in a rat model of T1D and the ability of insulin treatment to normalize these metabolic changes. These studies demonstrated that although insulin reversed a majority of the metabolic changes cause by diabetes, a “hypomethylated environment” develops in detrusor that is only partially reversed with glycemic control. We confirmed that this hypomethylated environment is associated with epigenetic changes in the detrusor genome, which are again mostly, but not completely, reversed with glycemic control. Overall our studies provide strong molecular evidence for a mechanism by which diabetes alters methylation status and patterns in the detrusor genome, leading to epigenetic modification and hyperglycemic memory, and suggest novel treatment strategies for diabetic patients who have attained glycemic control, but continue to experience DBD.

## 2 METHODS

Animals and tissues. All experimental protocols were approved by the Institutional Animal Care and Use Committee of the Albert Einstein College of Medicine. Sixteen male Fischer 344 rats were made diabetic for 3‐months by intraperitoneal injection with 35 mg/kg of streptozotocin (STZ) dissolved in citrate buffer (60 ml of 0.1 M Citric acid and 40 ml of 0.2 M Na_2_HPO_4_, pH 4.6), as previously described (Wang et al., 2016). Eight animals from this group of 3‐month diabetic animals were then treated daily with two units of long‐acting insulin (Lantus 100 units/mL, Sanofi‐Aventis, Bridgewater, NJ, USA) for one‐month. Eight non‐diabetic, age‐matched controls received an injection of vehicle (citrate buffer) and maintained for 3‐months. Rats were housed in individual cages and allowed free access to food and water. Blood glucose levels were determined with the OneTouch Ultra 2 Glucometer; Life Scan, Milpitas, CA); a reading of 250 mg glucose/dl for three consecutive days was considered indicative of hyperglycemia. Animals from each group were euthanized, bladders removed, and immediately placed into cold phosphate‐buffered saline (137 mM NaCl, 8 mM Na_2_HPO_4_, 2.7 mM KCl, and 1.47 mM KH_2_PO_4_, pH 7.4) and the detrusor manually separated from the mucosal. Detrusor tissue from five animals in each group that was to be used in the metabolomic analysis was snap‐frozen as previously described (Wang et al., 2016); tissue used in other molecular determinations was immediately processed to prepare genomic DNA or protein extracts.

Metabolomic analysis. Metabolomic analysis was performed by Metabolon, Inc as previously described (Wang et al., 2016). For data display purposes and statistical analysis, each metabolite was rescaled to set the median equal to 1. In addition, any missing values were assumed to be below the limits of detection, and these values were imputed with the compound minimum (minimum value imputation). Following median scaling and imputation of missing values, statistical analysis of log‐transformed data was performed using “R” (http://cran.r‐project.org/), which is a freely available, open‐source software package. Metabolites that differed significantly between the experimental groups were determined using ANOVA contrasts. *p* values ≤.05 were considered statistically significant, and *p* values <.10 were reported as trends. Multiple comparisons were accounted for by estimating the false discovery rate using *q* values.

DNA extraction and global DNA methylation analysis. Total genomic DNA was extracted from the detrusor of three animals from each group using the DNeasy Blood and Tissue Kit (Qiagen, Germantown, MD, USA) according to the manufacturer's instructions. Global DNA methylation was quantified using a Methylated DNA Quantification Kit (Colorimetric) (Abcam, Cambridge, MA, USA), following the manufacturer's protocol.

Epigenetic analysis. HELP‐tagging assay was performed by the Epigenomics Shared Facility at the Albert Einstein College of Medicine for massively parallel sequencing, essentially as previously described (Chamberlain et al., 2014). About 5 μg of extracted genomic DNA was digested to completion by either HpaII or MspI and the digested DNA was ligated to two custom adapters containing Illumina adapter sequences, an Ecop15I recognition site, and the T7 promoter sequence. Using Ecop15I, isolated sequence tags flanking the sites were digested by each enzyme, methylation‐sensitive HpaII or methylation‐insensitive MspI, followed by massively parallel sequencing of the resulting libraries (Illumina Technology). DNA methylation scores from 0 (fully methylated) to 100 (unmethylated) were filtered by confidence scores. These confidence scores were calculated for each sample based on the total number of HpaII‐generated reads as a function of the total number of MspI‐generated reads, excluding loci for which the confidence score is lower than the expected mean by locus. To understand the relative effects of known technical covariates acting on methylation data variability, principal component analysis (R package *princomp*) was performed on the DNA methylation score obtained from preprocessed data. Candidate differentially methylated loci are identified using analysis of variance with pairwise two‐tailed Turkey‐tests when comparing controls with either diabetes or insulin treatment as well as two‐sided *t* tests when comparing control/cases to define locus‐specific differences in average methylation between groups. We defined candidate differentially methylated loci to have a difference between mean DNA methylation scores >20% and a *p* value <.05.

Western blot analysis. Detrusor tissue was dispersed in lysis buffer (Cell Signaling Technology, Boston, MA, USA) following a standard protocol. Protein concentration was determined using a BCA protein assay kit (Thermo Fisher Scientific, Waltham, MA, USA) following the manufacturer's instructions. Equal amounts of proteins from each sample were separated on an SDS‐PAGE gel and transferred onto a PVDF membrane (Bio‐Rad, Hercules, CA, USA). Following blocking with PBS‐Tween‐20 containing 5% nonfat dry milk for 1 hr, membranes were incubated overnight at 4°C with primary antibody (anti‐NADH‐ubiquinone oxidoreductase 18 kDa subunit (Ndusf4), anti‐Phosphofructokinase (muscle) (Pfkm)), anti‐MaxiK potassium channel, α‐subunit (Kcnma1), anti‐Acyl‐coenzyme A dehydrogenase, Short/Branched Chain (Acadsb) anti‐Aldehyde dehydrogenase 3 family member A1 (Aldh3a)) and anti‐β‐Actin (Sigma‐Aldrich, St. Louis, MO, USA), followed by the secondary antibody labeled with horseradish peroxidase‐conjugated. Immunoreactive bands were detected by an enhanced chemiluminescence kit (PerkinElmer, Waltham, MA, USA). The intensities of the resulting bands were quantified using the software ImageJ 1.47q (U.S. National Institutes of Health, Bethesda, MD, USA). Data are shown as means ± SD unless otherwise indicated. Student's *t* test was performed using GraphPad Prism software (GraphPad Software Inc., San Diego, CA, USA). Significance was attributed to values of *p* < .05.

## 3 RESULTS

### 3.1 Insulin treatment reverses the majority of the effects of diabetes on the metabolome of bladder detrusor tissue

A total of 607 metabolites were detected in common at significant levels in 3‐month diabetic, 3‐month diabetic with 1 month‐insulin treatment and non‐diabetic, age‐matched controls (see Supplemental Table S1). As shown in Table 1, 171 of these 607 (28.2%) of these metabolites had significantly (*p* ≤ .05) changed levels in 3‐month diabetic compared with non‐diabetic, age‐matched controls (19.9% upregulated and 8.2% downregulated). One month of insulin treatment of animals with 3‐months of diabetes normalized the levels of a majority of these metabolites to those of non‐diabetic, age‐matched controls (112 of the 171 (65.5%), although insulin did cause change levels of an additional small number of metabolites (less than 6%). Glycemic control, therefore, reversed most, but not all, of the effects on the metabolome caused by hyperglycemia.

**Table 1:** Number of metabolites with altered levels in response to diabetes and its treatment with insulin

	Metabolites with a changed level in 3‐month diabetic compared to non‐diabetic, age‐matched controls.	Metabolites with a normalized level in 3‐month diabetic after 1‐month insulin treatment.	Metabolites with a changed level in response to insulin treatment compared to non‐diabetic, age‐matched control.
Metabolites at increased levels (*p* ≤ .05)	121/607 (19.9%)	80/121 (66.1%)	19/607 (3.1%)
Metabolites at increased levels (*p* ≤ .1)	156/607 (25.7%)	110/156 (70.5%)	36/607 (5.9%)
Metabolites at decreased levels (*p* ≤ .05)	50/607 (8.2%)	32/50 (64%)	9/607 (1.5%)
Metabolites at decreased levels (*p* ≤ .1)	69/607 (11.4%)	45/69 (65.2%)	17/607 (2.8%)

### 3.2 Insulin treatment reverses the effects of diabetes on energy‐generating biochemical pathways leading to oxidative stress and advanced glycation products

**Figure 1:**
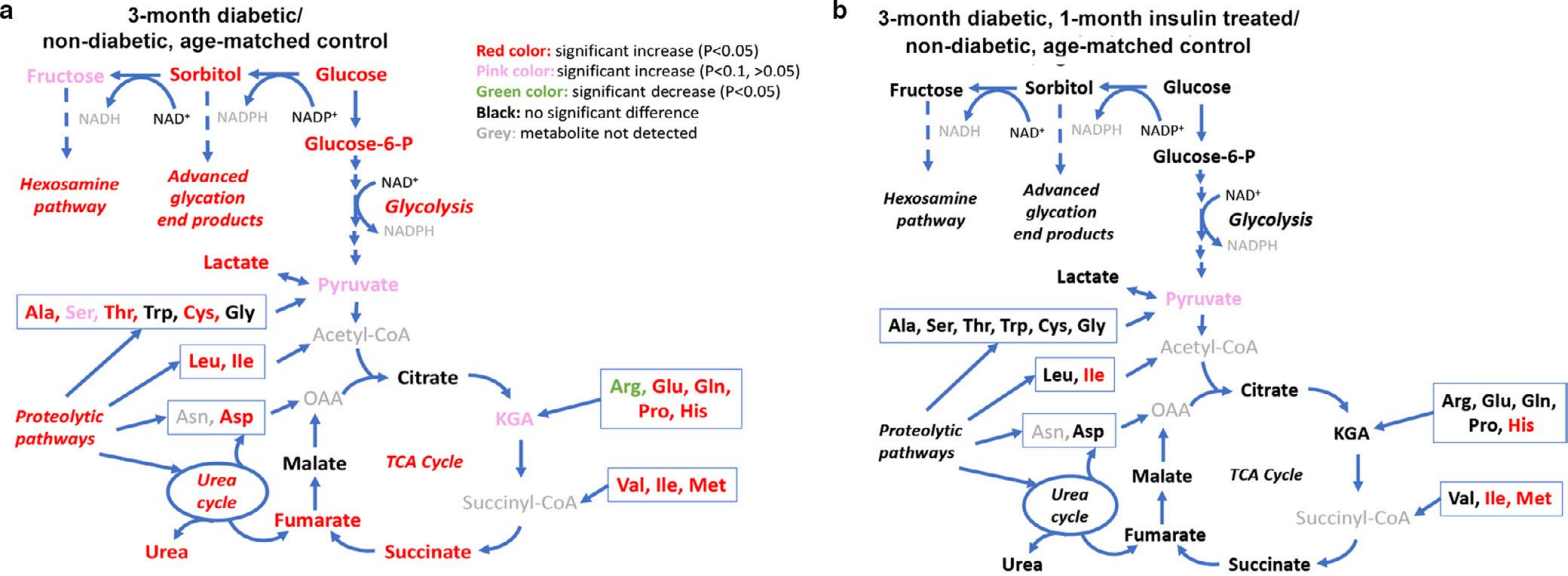
Comparison of changes in detrusor metabolite levels of major energy‐generating pathways between (a) 3‐month diabetic versus non‐diabetic, age‐matched controls and (b) 3‐month diabetic, 1‐month insulin‐treated versus non‐diabetic, age‐matched controls. For each animal group metabolite levels were determined in*N* = 5 animals. The following color code was used for a difference in metabolite level compared to the non‐diabetic, age‐matched control: red = fold‐change >1,*p* < .05; pink = fold‐change >1,*p* < .1; dark green = fold‐change <1,*p* < .05; black = no significant difference; grey = metabolite not detected

Overall, the changes in detrusor metabolism after 3‐months of diabetes were comparable to our prior publication which had investigated the changes in metabolism following one month of diabetes (Wang et al., 2016). For example, after 3‐months of diabetes there was significant (*p* < .05) altered levels of metabolic markers of diabetes, such as glucose (4.86‐fold increase), lactate (1.84‐fold increase), 2‐hydroxybutyrate (28.18‐fold increase), bile acids ﻿(for example, cholate (8.75‐fold increase), glycocholate (18.00‐fold increase), taurocholate (2.96‐fold)), as well as metabolites indicating the activation of branched‐chain amino acid metabolism, the pentose‐phosphate, glycolytic and polyol pathways (see Figure 1 and supplemental Table S1). However, there was evidence of some progressive changes in metabolism with prolonged diabetes; after 3‐months of diabetes there were significantly increased levels of TCA cycle metabolites than observed after 1‐month, suggesting an increase in the oxidative stress burden on detrusor tissue as diabetes progresses. However, if animals with 3‐months of diabetes were treated with one month of insulin then the levels of glucose in detrusor, as well as the majority of metabolites and metabolic pathways, were normalized and not significantly different to non‐diabetic, age‐match controls (see Figure 1 and Supplemental Table S1).

### 3.3 Prolonged diabetes results in a hypomethylated environment in detrusor tissue only partially reversed by insulin treatment

**Figure 2:**
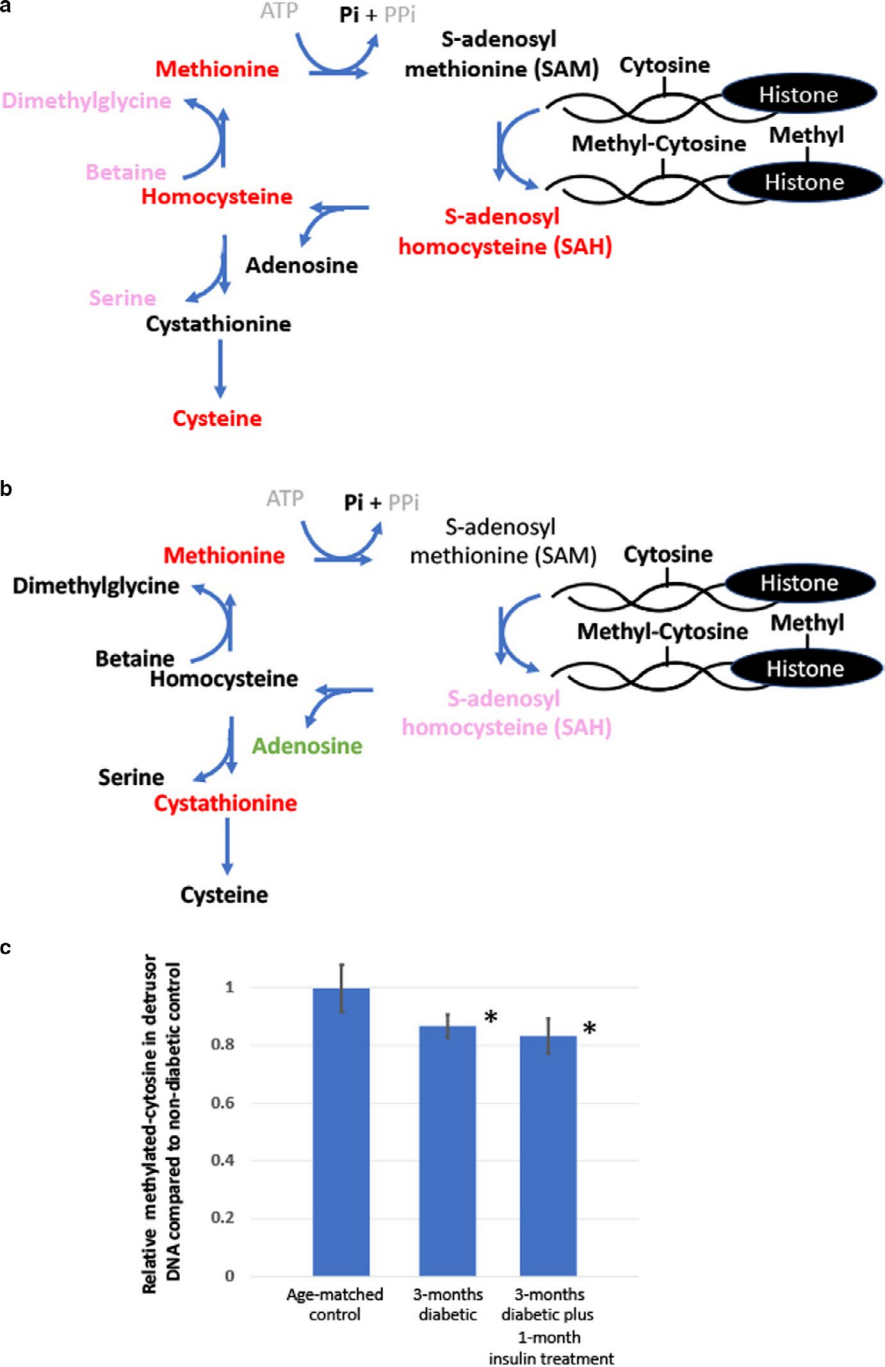
Comparison of changes in detrusor metabolite levels involved in trans‐sulfuration pathways that would be expected to impact the methylation status of cells methylation pathways between (a) 3‐month diabetic versus non‐diabetic, age‐matched controls and (b) 3‐month diabetic, 1‐month insulin‐treated versus non‐diabetic, age‐matched controls. For each animal group metabolite levels were determined in *N* = 5 animals. The following color code was used for a difference in metabolite level compared to the non‐diabetic, age‐matched controls: red = fold‐change >1, *p* < .05; pink = fold‐change >1, *p* < .1; dark green = fold‐change <1, *p* < .05; black = no significant difference; grey = metabolite not detected. C) Average relative methylated cytosine in detrusor DNA compared to non‐diabetic, age‐matched controls (*N* = 3, * = significant decrease in methylated DNA relative to non‐diabetic, age‐matched controls, *p*‐value < 0.05)

After 3‐months of diabetes, we found evidence that there were significant changes in transsulfuration pathways in detrusor tissue that would be expected to impact the methylation status of cells (Figure 2a,b, Supplemental Table S1). Interestingly, metabolic analysis of mucosal tissue from the same set of experimental animals provided no evidence of a change in the transsulfuration pathways that might affect methylation status (data not shown). The ratio of SAM/SAH has been frequently used as an indicator of the cellular methylation potential. In prior published studies, after 1‐month of diabetes, the SAM/SAH ratio in detrusor was 1.17, whereas after 3‐months of diabetes the ratio was 0.7, suggesting a progressively developing hypomethylation environment with the duration of diabetes. In 3‐month diabetic animals treated with insulin, the SAM/SAH ratio is not normalized and remains below 1 (0.55). This would suggest as diabetes progresses detrusor tissue would have decreased levels of methyl‐donor substrates.

In order to confirm the hypomethylation status, we used a commercial methylation DNA quantification kit to compare the relative levels of methylated‐cytosine (3‐MC) in detrusor genomic DNA (Figure 2c). We demonstrated that after 3‐months of diabetes the percentage of 3‐MC decreased by approximately 13%, and this decrease was not significantly normalized following 1‐month of insulin treatment.

### 3.4 Genome‐wide DNA‐methylation profiling demonstrates that diabetes results in epigenetic changes in the detrusor genome, the majority (but not all) of which are reversed by insulin treatment

We performed genome‐wide methylation profiling on DNA from the detrusor 3‐month diabetic, 3‐month diabetic with 1 month‐insulin treatment, and non‐diabetic age‐matched controls. An average of 1,171,938 (range 1,088,063–1,270,170) CpG loci were identified covering an average of 10.25% (range 5.05%–14.15%) of the entire chromosome (see supplemental Table S2). For comparison between the groups, only the CpG loci common to all samples (*n* = 65,980) were analyzed. After 3‐months of diabetes, a total of 1,696 (2.6%) of the identified loci were found to have altered methylation compared to age‐matched, non‐diabetic controls with the majority of these (1,389 (81.9%)) normalized by treatment with insulin (Supplemental Table S2).

**Figure 3:**
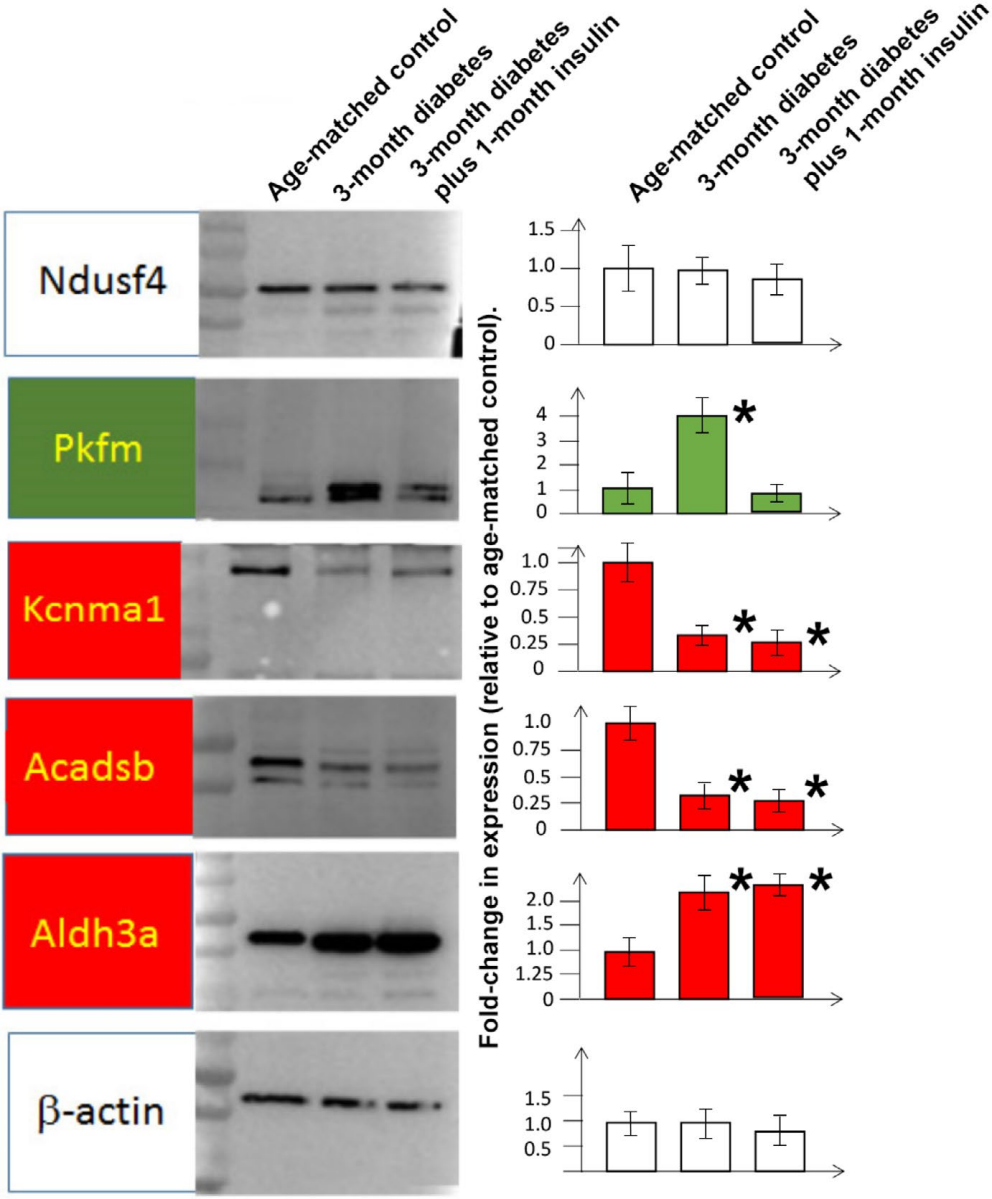
Protein expression in bladder detrusor in non‐diabetic, age‐matched controls, 3‐month diabetic, 3‐month diabetic plus one‐month insulin treatment. Left panel: Representative immunoblots are shown for proteins chosen based on the results in Supplemental Table S2 as representing genes in loci where the methylation pattern was changed in diabetic animals, but reversed (highlighted with green background), or not reversed (highlighted with red background), following insulin treatment. White background represents genes that are in loci where the methylation pattern was not changed with diabetes. Right panel: Bars represent the average fold‐change in protein expression compared to non‐diabetic, age‐match controls (*N* = 3 animals per group) determined by densitometric analysis of Western blots normalized to the housekeeping gene, β‐actin. * = significant difference (*p* < .05) in protein expression compared to non‐diabetic, age‐matched controls

To confirm that the methylation status reflected changes in the expression of proteins, we selected genes from Supplemental Table S2 which were in loci that did not undergo changes in methylation pattern with diabetes or following insulin treatment (e.g., NADH‐ubiquinone oxidoreductase 18 kDa subunit (Ndusf4)), underwent methylation pattern changes with diabetes that was reversible with insulin treatment (e.g., Phosphofructokinase (muscle) (Pfkm)) or was not reversed by insulin treatment (e.g., MaxiK potassium channel, α‐subunit (Kcnma1), Acyl‐coenzyme A dehydrogenase, Short/Branched Chain (Acadsb), and Aldehyde dehydrogenase 3 family member A1 (Aldh3a)) or a methylation pathway unchanged by diabetes or insulin treatment (white background). We determined protein expression by Western blot (normalized to β‐actin). As shown in Figure 3, the methylation patterns are predictive of changes in the expression of the selected proteins. For example, Pkfm expression is upregulated with diabetes, but normalized with insulin treatment. Acadsb has down‐regulated expression not reversed by insulin treatment and Aldh3a has up‐regulated expression with diabetes, not reversed by insulin treatment.

## 4 DISCUSSION

Our studies are the first to provide evidence of “hyperglycemic memory” in the bladder detrusor at the molecular/biochemical level. We extended our previous studies on the effects of 1‐month of diabetes (early‐stage) on detrusor metabolism (Wang et al., 2016) to 3‐months, representing a later‐stage diabetes early time point of diabetes. Comparing our previously published data on the metabolome of the detrusor of 1‐month diabetic animals, with the present study on 3‐month diabetic animals, we observed similar effects on metabolites, particularly those involved in pathways associated with the development of oxidative stress, although there was evidence that with the longer duration of diabetes these effects were increasing. In particular, after 3‐months of diabetes there was a decrease in the SAM/SAH ratio, which would be expected to correlate with a hypomethylated environment in detrusor tissue. We confirmed this by showing a decrease in the percentage of methylated cytosine in detrusor DNA and changes in the methylation pattern of at least 1,696 gene loci. Insulin treatment lowered blood and detrusor tissue glucose levels, which reversed a majority of the changes in metabolism caused by diabetes. However, several metabolites remained unchanged, including the SAM/SAH ratio suggesting a continuing hypomethylated state. This was reflected by a failure of glycemic control to significantly reverse the percentage of methylated cytosine in detrusor DNA or the epigenetic changes in the methylation pattern of 307 gene loci. Overall, our work suggests a molecular mechanism for hyperglycemic memory in the diabetic detrusor through the epigenetic modification of genomic DNA resulting from an environment of hypomethylation, that persists even after the normalization of tissue glucose levels.

Although urodynamic determinations were not performed as part of this study, a large‐scale study published by our laboratory in 2009 (Melman et al., 2009) investigated the effects of diabetes and insulin treatment on bladder function in the same rat model of T1D used here. This study showed that although insulin treatment normalized several cystometric parameters to those of non‐diabetic, age‐matched controls, it was not effective in restoring bladder compliance, voids per hour, and spontaneous activity. Therefore, glycemic control by insulin in this animal model of T1D can only be considered to be partially effective in normalizing the bladder function. We hypothesize that the failure to reverse all the pathophysiological effects in the diabetic bladder is a result of hyperglycemic memory, such that even with glycemic control, metabolic pathways responsible for this bladder dysfunction are not normalized.

SAM is a metabolic product of methionine and ATP generated through the enzymatic action of methionine‐adenosyltransferase (( Williams & Schalinske, 2008) and see Figure 2). SAM is then utilized as a methyl‐donor of several substrates, including DNA, generating SAH as a by‐product which is subsequently hydrolyzed to homocysteine. This reaction, catalyzed by SAH hydrolase is reversible, during which process increased homocysteine levels can lead to the accumulation of SAH, a strong feedback inhibitor of SAM‐dependent DNA methyltransferases and therefore an inhibitor of the transmethylation reactions. A decrease in the SAM/SAH ratio would, therefore, represent a reduced methylation index in cells, and it has been previously reported that diabetics with nephropathy have a decreased lymphocytic SAM/SAH ratio and decreased transmethylation flux (Poirier et al., 2001; Tessari et al., 2005).

The SAM/SAH ratio has been well‐established to correlate with the methylation status of DNA (Caudill et al., 2001). Methylation of DNA is considered to play a major role in epigenetic mechanisms associated with diabetic complications and hyperglycemic memory (Reddy et al., 2015). Epigenetic changes can have a major effect on gene regulation and thereby biological processes and pathophysiology (Kouzarides, 2007; Portela & Esteller, 2010). Our studies confirmed that in the detrusor there were changes in methylation patterns of approximately 2.6% of genes where CpG were detected, but even after 1‐month of insulin treatment, 18.1% of genes did not return to the methylation pattern seen in non‐diabetic detrusor. We confirmed that at least in the small number of genes we investigated, that the change in methylation patterns at specific loci corresponded with protein expression from the genes encoded in these loci. This group included Kcnma1, which encodes the pore‐forming α‐subunit of the MaxiK potassium channel. Because of the involvement of the MaxiK gene in regulating detrusor tone (and thereby bladder relaxation/contractility), it has been long‐proposed as a pharmacologic target for detrusor over‐activity (Petkov, 2014; Siemer et al., 2000; Wang et al., 2014). As shown in Figure 3, we were able to validate that expression Kcnma1 correlates with the changes seen in methylation patterns, that is, the change in methylation pattern occurring with diabetes is not reversed through insulin treatment, and the down‐regulation of Kcnma1 with diabetes persists even with insulin treatment. Although the expression and activity of Kcnma1 in the bladder is known to be decreased with diabetes, our data are the first to suggest this decrease is not reversed by glycemic control. This result has implications for the use of Kcnma1 as a target for DBD in patients with glycemic control. Since the MaxiK α‐subunit expression is low with diabetes, and this is not reversed with insulin treatment, the proposed use of pharmacologic MaxiK channel openers would either have to be used at much higher doses than when used in non‐diabetics to obtain the same physiological effect, or, the level of target is so low, that attempts to increase activity through the use of pharmacologic agents would have no effect on physiology. However, gene therapy, for example, using genetic vectors overexpressing MaxiK from foreign, unmodified epigenomic DNA (which have been investigated in clinical trials for safety (Melman et al., 2007; Rovner et al., 2020), would be expected to be effective. Overall, although our epigenetic studies covered only 7% of the complete genome, it supports an approach where epigenomic data can be used to identify “actionable targets” to treat DBD; changes in protein expression occurring with diabetes that are not normalized by insulin treatment represent the most likely targets for treating pathophysiology associated with hyperglycemic memory (Reddy et al., 2015).

Our evidence for hyperglycemic memory in the bladder also provides a rationale for the treatment of diabetic patients who have achieved glycemic control, but have recalcitrant DBD, through approaches that target the methylation index in detrusor tissue. For example, there is evidence that dietary supplementation with Methionine prevents diabetes‐induced epigenetic alterations, and therefore it has been proposed that regulating dietary Methionine levels could be therapeutically exploited for the treatment of metabolic diseases (Navik et al., 2019). Another approach might be to use drugs that are known to affect the methylation index of tissues. For example, the anti‐diabetic biguanide metformin has been shown to promote global DNA methylation in non‐cancerous, cancer‐prone, and metastatic cancer cells by decreasing SAH level (which as described above is a strong feedback inhibitor of SAM‐dependent DNA methyltransferase), while promoting the accumulation of SAM (Cuyàs et al., 2018). Therefore, metformin might be used to modulate the methylation index in detrusor caused by T1D and reverse epigenetic changes in the genome (Cuyàs et al., 2018).

Conclusions. We provide the first evidence of “hyperglycemic memory” in the bladder detrusor at the molecular/biochemical level, focusing on a changed methylation index of cells occurring with diabetes. The failure of glycemic control to reverse the hypomethylation environment of the detrusor cell results in persistent epigenetic changes associated with irreversible changes in metabolism and gene expression. Our work suggests novel approaches to treat recalcitrant DBD in patients who are under glycemic control. For example, pharmacological approaches that target the hypomethylation environment established in the diabetic detrusor or the use of epigenomic data to identify “actionable therapeutic targets”; genes in loci which diabetes results in modulated methylation patterns and protein expression that are not normalized by insulin treatment and are therefore the most likely targets for hyperglycemic memory.

## CONFLICT OF INTEREST

No conflicts of interest, financial or otherwise, are declared by the authors.

## AUTHOR CONTRIBUTIONS

Y.W. and K.P.D. conception and design of research; Y.W. and M.T.T. performed experiments; Y.W. and K.P.D. analyzed the data; K.P.D. prepared figures; K.P.D. drafted the manuscript; Y.W., M.T.T., and K.P.D. edited and revised the manuscript; K.P.D. interpreted the results of experiments; K.P.D. approved the final version of the manuscript.

## Supporting information



Table S1

Table S2
